# Intracellular Pathways of Holothuroid Oocyte Maturation Induced by the Thioredoxin Trx-REES

**DOI:** 10.3390/antiox10081201

**Published:** 2021-07-27

**Authors:** Jérôme Delroisse, Aline Léonet, Henri Alexandre, Igor Eeckhaut

**Affiliations:** 1 Biology of Marine Organisms and Biomimetics Unit, Research Institute for Biosciences, University of Mons, 7000 Mons, Belgium; Aline.Leonet@heh.be; 2 Belaza Marine Station, Institut Halieutique et des Sciences Marines, University of Toliaria, Toliaria 601, Madagascar; 3 Haute Ecole Du Hainaut, 7000 Mons, Belgium; 4 Embryology Laboratory, Research Institute for Biosciences, University of Mons, 7000 Mons, Belgium; Henri.Alexandre@umons.ac.be

**Keywords:** sea cucumber, oocyte, maturation, Trx-REES, DTT, *Holothuria scabra*, *Holothuria tubulosa*

## Abstract

In holothuroids, oocyte maturation is stopped in ovaries at the prophase I stage of meiosis. In natural conditions, the blockage is removed during the spawning by an unknown mechanism. When oocytes are isolated by dissection, the meiotic release can be successfully induced by a natural inducer, the REES (i.e., Rough Extract of Echinoid Spawn) that is used in aquaculture to obtain viable larvae in mass. A thioredoxin has recently been identified in the REES as the molecule responsible for holothuroid oocyte maturation. As a redox-active protein, thioredoxin is thought to reduce target proteins within the oocyte membrane and initiate an intracellular reaction cascade that leads to the unblocking of the oocyte meiosis. Our results allow us to understand additional steps in the intracellular reaction cascade induced by the action of thioredoxin on oocytes. Pharmacological agents known to have activating or inhibiting actions on oocyte maturation have been used (Forskolin, Isobutylmethylxanthine, Hypoxanthine, 6-dimethyaminopurine, Lavendustin, Genistein, Roscovitine, Cycloheximide). The effects of these agents were analysed on oocytes of the holothuroid *Holothuria tubulosa* incubated with or without REES and were compared to those obtained with another reducing agent, the dithiothreitol. Our results demonstrated that, at the opposite of dithiothreitol-induced oocyte maturation, thioredoxin-induced oocyte maturation is cAMP independent, but dependent of the presence of calcium in the seawater. Both pathways of induction require the activation of protein serine/threonine kinases.

## 1. Introduction

Oocyte maturation refers to a release of the meiotic arrest that allows oocytes to shift from prophase I to fertilisation. This process is relatively well studied in the animal kingdom, where fertilisation occurs at various stages of meiotic divisions. In echinoderms, asteroid oocytes are mature at metaphase I [1] while echinoid [2], ophiuroid [3], crinoid [4] and holothuroid [5] oocytes are mature at the ootid stage in metaphase II.

**Figure 1:**
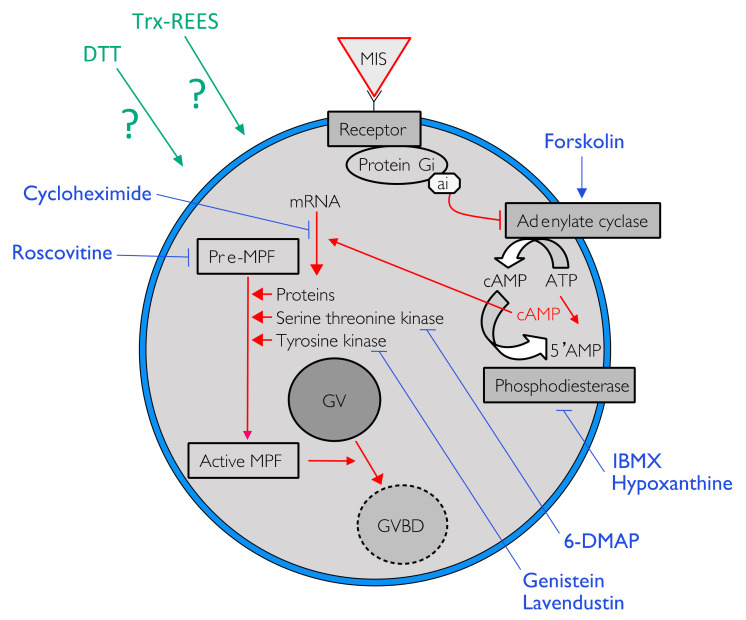
One of the possible pathways to induce oocyte maturation. Signal in red indicated the cytoplasmic pathway activated by the liaison of the maturation-inducing substance (MIS) on his receptor to trigger the resumption of meiosis by the activation of the maturation-promoting factor (MPF). Blue lines indicate action sites of various pharmacological agents tested to inhibit the resumption of oocyte maturation.

During the course of oogenesis, the meiosis is stopped in all animals at prophase I of meiosis (i.e., germinal vesicle stage). It is well known that the progression from prophase I to the maturation stage is initially promoted by gonad-stimulating substances (GSS) whose molecular nature varies from one taxon to another. This gonad-stimulating substance induces the production of maturation-inducing substance (MIS) in ovarian follicle cells. The action of the maturation-inducing substance together with its receptor located on the oocyte plasma membrane is to transfer the signal into the cytoplasm where the maturation-promoting factor (MPF) is activated (Figure 1). The maturation-promoting factor is considered identical for all eukaryotes. It has been identified as being a heterodimer composed of a catalytic subunit, the p34 protein, and a regulatory subunit, the cyclin B (in *Xenopus* [6]; in *Asterias* [7]). The maturation-promoting factor has been shown to induce nuclear envelope breakdown, chromosome condensation and spindle formation [8]. In the literature, the information currently available on the cytoplasmic mechanisms controlling the oocyte maturation in echinoderms mainly concerns asteroids where the gonad-stimulating substance, the maturation-inducing substance and the maturation-promoting factor are thus relatively well known. The asteroid gonad-stimulating substance is a neuropeptide—a member of the relaxin-type peptide family [9,10,11]—released from radial nerve [11,12,13,14], their maturation-inducing substance is the 1-Methyl Adenine (1MeAd, [15,16]) and the maturation-promoting factor is the complex made of p34^cdc2^ (CDK1 kinase) with the cyclin B [17].

In holothuroids, two peptides were isolated from the radial nerves of various species and acted on oocyte maturation, being thus potential gonad-stimulating substances. The first molecule isolated from five species of holothuroids was a peptide of a few thousand Daltons [5]. Later, a peptide of 4.8 kDa was isolated from the radial nerve of *Apostichopus japonicus*. It induces germinal vesicle breakdown (GVBD) in 80% of oocytes [18]. At the same time, Kato et al. purified and identified another smaller peptide named cubifrin (NGIWYamide), also produced by the radial nerve of *A. japonicus* [19]. Cubifrin induces oocyte maturation, ovulation and spawning. A synthetic derivative, cubifrin-L (NGLWYamide), was shown to be even more active than the natural product [20]. The cubifrin peptide has also been discovered in the transcriptomes of *Holothuria scabra* and *H. glaberrima* [21]. Chayamoon et al. characterised the cubifrin in *H. scabra* and further investigated its effect on spawning [22]. The deduced amino acid sequence of the neuropeptide precursor transcript provided several copies of two different mature peptide sequences designated cubifrin-Y and cubifrin-F [22]. In situ hybridisations showed intense expression of cubifrin transcript within the wall of the ovarian tubule at late ovarian stages, suggesting that cubifrin is produced locally and probably stimulated the spawning by causing contraction of the wall of the ovarian tubule [22]. Yet, Chieu et al. successfully produced a recombinant of a neurohormone relaxin-like gonad-stimulating peptide (RGP) of *H. scabra* (homologous to the sea star gonad-stimulating substance) from the yeast *Pichia pastoris* [23]. The purest form induced 98.56% of germinal vesicle breakdown in *H. scabra* [23]. Upon single injection into females of *H. scabra*, the recombinant relaxin-like gonad-stimulating peptide induced the spawning posture (i.e., head waving behaviour) followed by spawning within 90–170 min.

No maturation-inducing substance has currently been identified in holothuroids, but some authors have shown the importance of molecules acting on redox potential of the oocytes to induce maturation. Kishimoto and Kanati first demonstrated that the asteroid maturation-inducing substance, 1-Methyl Adenine, is not active on holothuroid oocytes maturation, but they highlighted that incubation of *A. japonicus* oocytes in the reducing agent dithiothreitol (DTT) alone had a slight effect on the induction of maturation of *A. japonicus* oocytes [24]. Yet, *A. japonicus* oocytes which had been treated with pronase did undergo the germinal vesicle breakdown following dithiothreitol treatment, although pronase by itself was ineffective in inducing the germinal vesicle breakdown [24]. They suggested that reduction of disulfide bonds at the oocyte surface induces the germinal vesicle breakdown and subsequent meiotic process in holothuroid oocytes. They also suggested that the unidentified holothuroid maturation-inducing substance brings about an increase in protein-SH content of oocyte cortex, as is the case with asteroid oocytes stimulated by 1-Methyl Adenine. In two other holothurian species, *H. leucospilota* and *H. pardalis*, Maruyama showed that DTT treatment of denuded oocytes led to maturation and subsequent normal fertilisation and embryonic development [25]. Later, with the use of pharmacological agents, Karaseva and Khotimchenko demonstrated that the reduction induced by DTT at the oocyte surface leads to a decrease in the intracellular cAMP level of *A. japonicus* oocytes, cAMP acting as a secondary messenger in DDT-induced maturation of holothuroid oocytes [26].

Our laboratory found that a raw extract of echinoid spawns (REES) efficiently induced holothuroid oocyte maturation [27]. REES is currently used in aquaculture to obtain mature oocytes which can then be fertilised in vitro and give embryos in large quantities throughout the year [28]. We have thereafter shown that the active molecule in REES was a thioredoxin (Trx-REES) and that Trx-REES is an effective holothuroid maturation inducer [27,28]: when applied to *Holothuria tubulosa* oocytes, it ensures both maturation (>90% in Trx-REES and DTT) and fertilisation of oocytes (>90% in Trx-REES; 40% with the DTT) [27]. Thioredoxins are small and multifunctional proteins containing a conserved CGPC (Cys-Gly-Pro-Cys) redox catalytic site [29]. The catalytic reaction of thioredoxins can be seen as a transfer of the disulfide bond from a substrate protein to the thioredoxin [29]. Organisms have developed their specialised subset of thioredoxins, which are located in various cellular compartments: some are abundant in the cytosol, others are translocated to the nucleus or mitochondria, associated with the cell membrane or secreted to the extracellular environment [29]. In mammals, animal thioredoxin family members include Trx-1 (12 kDa), Trx-2 (18 kDa), and a Trx-like protein [30]. Trx-REES is 18 kDa (similar to the mammalian Trx-2) [28]. Commercial Trx from *E. coli* was able to induce holothuroid oocyte maturation with similar percentages to the ones obtained when we used REES [28]. We also demonstrated that the synthetic catalytic site of the Trx-REES, WCNPCK, induced well the oocyte maturation of holothuroids [28].

The present paper analysed the intracellular pathway of the Trx-REES-induced oocyte maturation and compares it with the DDT-induced oocyte maturation. To understand the effects of these two types of inducers, we studied the effects of DTT, REES and Trx from *E. coli* with various pharmacological agents on the oocytes of *H. tubulosa.* The selected pharmacological agents are specifically affecting different intracellular pathways known to be important in animal oocyte maturation: influencing the level of cAMP (i.e., forskolin, 3-isobutyl-1-methylxanthine and hypoxanthine) and inhibiting the kinase activity (i.e., 6-dimethyl-aminopurine, lavendustin, genistein and roscovitine). We also analysed the potential role of extracellular calcium in the resumption of meiosis in *H. tubulosa* and *H. scabra* induced by the DTT and Trx-REES.

## 2. Materials and Methods

### 2.1. Sample Collection

Experiments were mainly conducted at the University of Mons (UMONS, Belgium), on the Mediterranean sea cucumber, *Holothuria tubulosa* Gmelin, 1788. Individuals of this species were collected by scuba diving in the natural reserve of Banyuls-sur-Mer (Banyuls-sur-Mer, France), and were sent to the UMONS laboratory (closed circuit seawater aquaria). *H. tubulosa* is the most frequent sea cucumber species in the western Mediterranean Sea.

Experiments concerning the effects of calcium of seawater were also conducted on the tropical sea cucumber *Holothuria scabra* Jaëger, 1833. The experiments involving *H. scabra* were carried out at the Polyaquaculture Research Unit (IH.SM; Toliara; Madagascar). Individuals were hand-collected at low tide in the sea grass beds of the Great Reef of Toliara. Living specimens were kept in aquaria supplied with circulating sea water. *H. scabra* is an Indo-Pacific species with a high commercial value. This species is classically targeted in sea cucumber aquaculture projects (e.g., [31,32]).

### 2.2. Pharmacological Induction of Oocyte Maturation

The induction of maturation activity of REES and DTT were measured on holothuroid oocytes that were obtained from the ovaries of dissected *H. tubulosa* individuals. The ovaries were isolated and washed several times with filtered sea water (FSW), then cut into pieces to release oocytes from gonadal tubules. The oocytes were first separated from the tubule fragments on a nylon sieve (200 µm mesh), then washed three times with FSW on a smaller sieve (100 µm mesh). Isolated oocytes were incubated in Petri dishes (concentration: 100 oocytes/mL) in the presence of various inductive molecules at different concentrations to test their effect on oocyte maturation. The inductive molecules were dithiothreitol (DTT) (10^−2^ M) (43815, Sigma-Aldrich, St. Louis, MO, USA), thioredoxin from *Escherichia coli* (0.12 mg mL^−1^) (T0910, Sigma-Aldrich, corresponding NCBI ID: AAA24693.1) or rough extract of echinoid spawn (REES). The REES was prepared at the Polyaquaculture Research Unit as in [28]. Regular sea urchins, *Tripneustes gratilla* (Linnaeus) 1758, were collected by hand at low tide in the sea grass beds of the Great Reef of Toliara. Ovaries were extracted from *Tripneustes gratilla* with fine forceps, rinsed with FSW and centrifuged at 5000 rpm (revolutions per min) for 2 × 10 min. Only the pellet was recovered, then was frozen, lyophilised (24 h) and reduced into powder. To obtain active fractions of this extract, the powder was dissolved in seawater in appropriate concentration (2 mg mL^−1^) and filtered on Whatman paper before use [28].

Intracellular pathway of the REES-induced maturation and DTT-induced maturation were studied in using eight pharmacological agents in appropriate concentrations (Table 1: active and non-toxic concentrations). For each pharmacological agent, a range of concentrations was first tested according to their effects mentioned in the literature (Table 1). At higher concentrations the pharmacological agent became toxic for oocytes or were not soluble. The smallest active and non-toxic concentration were thus used during the present experiments (Table 1). Forskolin, 3-isobutyl-1-methylxanthine (IBMX) and hypoxanthine were used to study the effect of REES and DTT on intracellular cAMP. The first is an activator of adenylate cyclase (AC) that transforms ATP into cAMP. The two last inhibits phosphodiesterase that cleaves cAMP in 5′AMP, which leads to a rapid decrease of the cAMP signal (Figure 1). Roscovitin is an inhibitor of CDK1 (the MPF is the complex made of p34^cdc2^, a CDK1 kinase, with the cyclin B) and 6-DMAP is an inhibitor of serine threonine kinase protein (p34 has a serine threonine kinase activity) (Table 1; Figure 1). Lavendustin and genistein are both inhibitors of tyrosine kinases useful in maturation of some pre-MPF and cycloheximide is an inhibitor of protein synthesis (Table 1; Figure 1).

The maturation of the oocytes was monitored by observing (i) the germinal vesicle breakdown (GVBD) and (ii) the formation of polar bodies with a light microscope after an incubation time of 2 h. Each test was made in three biological replicates (i.e., oocytes were collected from three different *H. tubulosa/H. scabra* individuals during three distinct experiments). The portion of mature oocytes was determined by randomly counting from 100 to 150 oocytes per sample. However, mature oocytes can be observed at any time in the ovaries of sea cucumbers in various proportions, these proportions varying according to the individuals and the season. This proportion of naturally matured oocytes is closer to 0 outside the spawning season and close to 100 when the individuals are ready to spawn. In all experiments, to record the effect of used inducers, we thus determined the percentage of spontaneous maturation present in placing extracted oocytes in FSW without any additives (i.e., the controls).

To determine the importance of seawater Ca^2+^ on holothurian oocyte maturation, some experiments were performed with artificial sea water (ASW) without Ca^2+^ (composition as follows: NaCl 445 mmol L^−1^, MgCl_2_ 60 mmol L^−1^, KCl 10 mmol L^−1^, NaHCO_3_ 2.4 mmol L^−1^, Hepes 10 mmol L^−1^ and EGTA 2.5 mmol L^−1^). Sea cucumber oocytes were placed into ASW without Ca^2+^ with REES or DTT. After an incubation time of 2 h, the maturation of the oocytes was monitored and compared to a control in FSW.

Furthermore, to assess whether follicle cells are important in REES-induced oocyte maturation, we tested REES on follicle-enclosed and on follicle-free oocytes. Full-grown oocytes taken from the holothurian ovaries were separated depending on whether they were follicle-enclosed and follicle-free (85 and 74 oocytes, respectively). This procedure was performed by gently pipetting using mouth-controlled micropipette. Mechanically defolliculated oocytes or follicle-enclosed oocytes were then incubated in a solution of REES 2‰ in filtered sea water. Control consisted of similarly prepared material not treated with REES.

### 2.3. Data Analyses

As the multiple tests were made on various *H. tubulosa/H. scabra* individuals, and where ovaries are not necessarily at the same stage of maturation, we standardised the results in the form of a maturation index (MI) [28]. The MI value was the percentage of mature oocytes observed in the various solutions (with or without pharmacological agents) divided by the number of mature oocytes in a positive control multiply by 100 as presented in the following formula: [MI = (MX)/(MC)] * 100, where MI is the value of the maturation index expressed in %; MX, the number of mature oocytes observed in the various solutions; MC, the number of mature oocytes in a positive control. When working with REES, the number of oocytes that matured in REES was the positive control; when working with DTT, the number of oocytes that matured in DTT was the positive control. With this transformation the MI in REES (versus in DTT) was always equal to 100%.

One-way ANOVA statistical analyses (multiple comparisons), followed by Dunnet post hoc tests or Tukey post hoc tests, and *t*-tests (pairwise comparisons) were performed using GraphPad Prism 5.0 on raw maturation rate data. *p* < 0.05 indicates statistical significance.

### 2.4. Animal Ethics

Collections were carried out in accordance with local and international laws. No special permits are needed for the marine invertebrate species used in this work, and no ethic approvals are required for this study because research on echinoderms is not subject to ethics regulation. The animals used in our experiments were maintained and treated in compliance with the guidelines specified by the Belgian Ministry of Trade and Agriculture.

## 3. Results

The percentage of sea cucumber oocytes that were mature before experiments was from 9 to 29%. Oocyte maturation with DTT or REES varied greatly according to the period and/or the used individual: it was, respectively, from 54–94% for the DDT and 48–94% for the REES. To assess the importance of follicle cell in the oocyte maturation induction, we tested REES on follicle-enclosed oocytes and on follicle-free oocytes. The MIs obtained were of 100% ± 2.1 for follicle-enclosed oocytes and 100% ± 3.2% for defolliculated oocytes. There is no significant difference between these two tests meaning that REES does not act on follicle cells.

**Figure 2:**
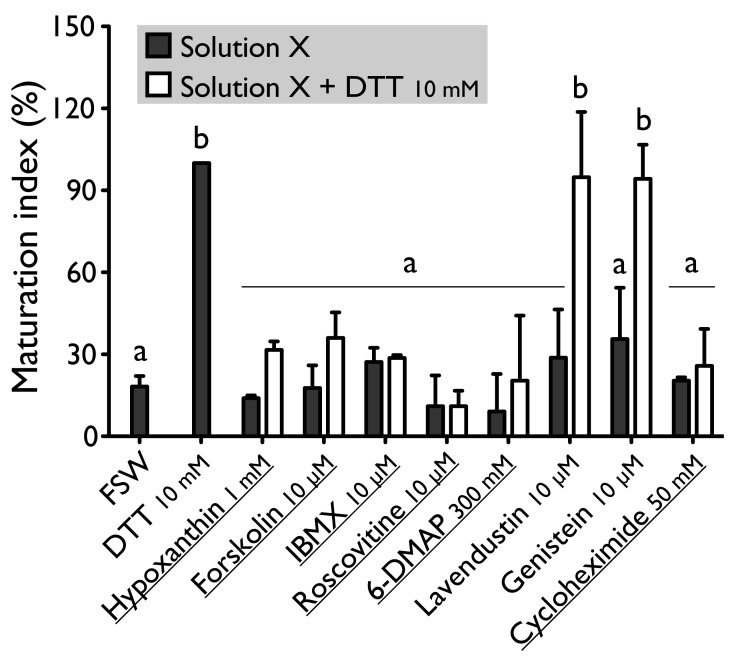
Effect of hypoxanthine (1 mM), forskolin (10 µM), IBMX (10 µM), roscovitine (10 µM), 6-DMAP (300 mM), lavendustin (10 µM), genistein (10 µM) and cycloheximide (50 mM) on *H. tubulosa* oocyte maturation, induced by dithiothreitol (DTT, 10 mM). Values are means of Maturation Index ± SD (*n* = 3 individuals). Columns surmounted by sign (**a**) or (**b**) are significantly similar to control (SW or DTT) surmounted with the same sign (P_Dunnett_ ≥ 0.05). For underlined solutions, the indices of maturation were not significantly different with or without DTT (P*_t_*_-test_ ≥ 0.05).

**Figure 3:**
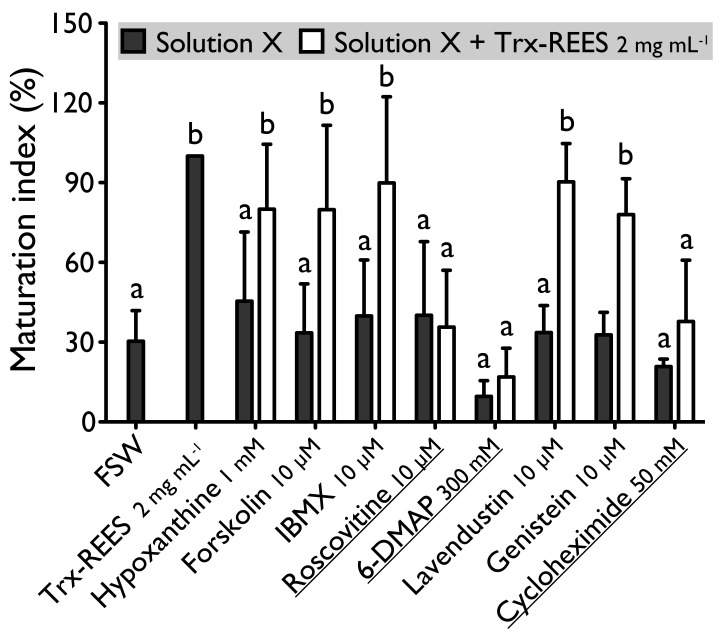
Effect of hypoxanthine (1 mM), forskolin (10 µM), IBMX (10 µM), roscovitine (10 µM), 6-DMAP (300 mM), lavendustin (10 µM), genistein (10 µM) and cycloheximide (50 mM) on *H. tubulosa* oocyte maturation, induced by Trx-REES. Values are means of Maturation Index ± SD (*n* = 3 individuals). Columns surmounted by sign (**a**) or (**b**) are significantly similar to control (SW or Trx-REES) surmounted with the same sign (P_Dunnett_ ≥ 0.05). For underlined solutions, the indices of maturation were not significantly different with or without Trx-REES (P*_t_*_-test_ ≥ 0.05).

Forskolin, known as an activator of adenylate cyclase (Figure 1), has been proven to usually suppress the oocyte maturation. On *H. tubulosa*, forskolin, used at the concentration of 10 µM, maintain oocyte in GVBD despite the presence of DTT (Figure 2). On the other hand, used at the same concentration, forskolin does not block oocyte maturation induced by REES (Figure 3). The MI obtained for oocytes treated with a solution containing forskolin-REES (79.8% ± 31.7%) and MI obtained for oocytes treated with REES (100%) were not significantly different (Figure 3). In the case of DTT, the MI reached 36.0% ± 9.4% for the solution forskolin-DTT (Figure 2). This rate was not significantly different to the MI of the negative control (i.e., FSW; 18.0% ± 3.8%) (P_Dunnett_ ≥ 0.05).

Hypoxanthine and IBMX inhibit phosphodiesterases (Figure 1), that causes an increase of the cAMP level in oocytes and so the blockage of the meiotic maturation (Figure 1). These two drugs suppress the DTT-induced oocyte maturation in *H. tubulosa* at respective concentrations of 1 mM and 10 µM (Figure 2). The MI in hypoxanthine-DTT (31.6% ± 3.1%) and in IBMX-DTT (28. 7% ± 1.0%) were significantly similar to the MI obtained with FSW (18.0% ± 3.9%) (Figure 2). However, REES effectively induces oocytes maturation despite the presence of hypoxanthine (1 mM) and IBMX (10 µM). In this case, the MI obtained for the solution hypoxanthine-REES (80.0% ± 24.5%) and IBMX-REES (89.9% ± 32.5%) were significantly similar to the MI obtained with oocytes treated with REES (100%) (Figure 3).

Implication of MPF, in sea cucumber oocyte maturation, can be controlled by use of roscovitine. It is a specific inhibitor of p34^cdc2^, the cyclin-dependent kinase component of MPF (Figure 1). DTT and REES were not efficient in presence of roscovitine (10 µM) (Figure 2 and Figure 3). The MI in roscovitine-DTT (11% ± 5.7%) and in roscovitine-REES (35.7% ± 21.3%) remained not significantly different to the sea water controls with DTT (18.0% ± 3.9%) or REES (30.3% ± 11.5%).

A concentration higher than 300 mM of 6-DMAP blocks serine threonine kinases (Figure 1) and suppresses the DTT-induced oocyte maturation (Figure 2). The use of the 6-DMAP showed that kinases were also required for meiosis resumption induced by REES (Figure 3), such as for DTT (Figure 2). The MI for 6-DMAP-DTT (20.3% ± 23.8%) and 6-DMAP-REES (16.9% ± 10.8%) treatments remained significantly the same than the sea water controls with DTT (18.0% ± 3.9%) or REES (30.3% ± 11.5%).

Lavendustin (10 µM) and genistein (10 µM), two drugs that inhibit tyrosine kinases (Figure 1), do not suppress either DTT nor REES effect (Figure 2 and Figure 3). Indeed, the MI obtained with these inhibitors and DTT or REES were close to MI of positive control: the MI in solution of lavendustin-DTT was of 94.8% ± 23.9% and in genistein-DTT of 94.3% ± 12.5% (Figure 2); the MI in solution of lavendustin-REES was of 90.3% ± 14.4% and in genistein-REES of 78.0% ± 13.5% (Figure 3).

Cycloheximide (50 mM), a protein synthesis inhibitor (see Figure 1), was found to produce the same effect that the roscovitine (10 μM) and the 6-DMAP (300 mM), oocytes stay blocked in prophase I despite the presence of DTT or REES (Figure 2 and Figure 3): the MI with cycloheximide-DTT was of 25.8% ± 13.5% and with DTT of 18.0% ± 3.9%; the MI with cycloheximide-REES was of 37.8% ± 13.5% and with REES of 30.3% ± 11.5%.

**Figure 4:**
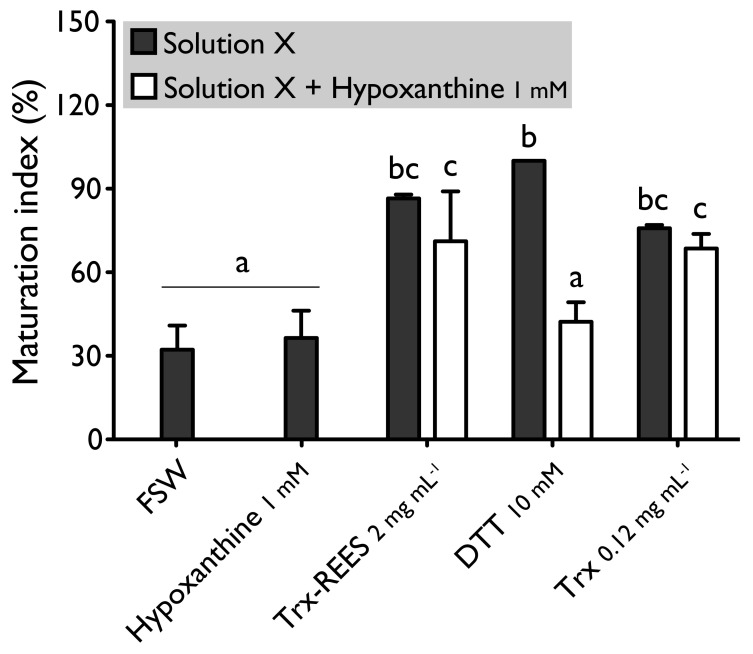
Effect of hypoxanthine (1 mM), on *H. tubulosa* oocyte maturation, induced by Trx-REES (2 mg mL^−1^), DTT (10 mM) or thioredoxin from *E. coli* (Trx, 0.12 mg mL^−1^). Values are means of maturation index ± SD (*n* = 3 individuals). Means sharing at least one letter are not significantly different (T_Tukey_ ≥ 0.05).

**Figure 5:**
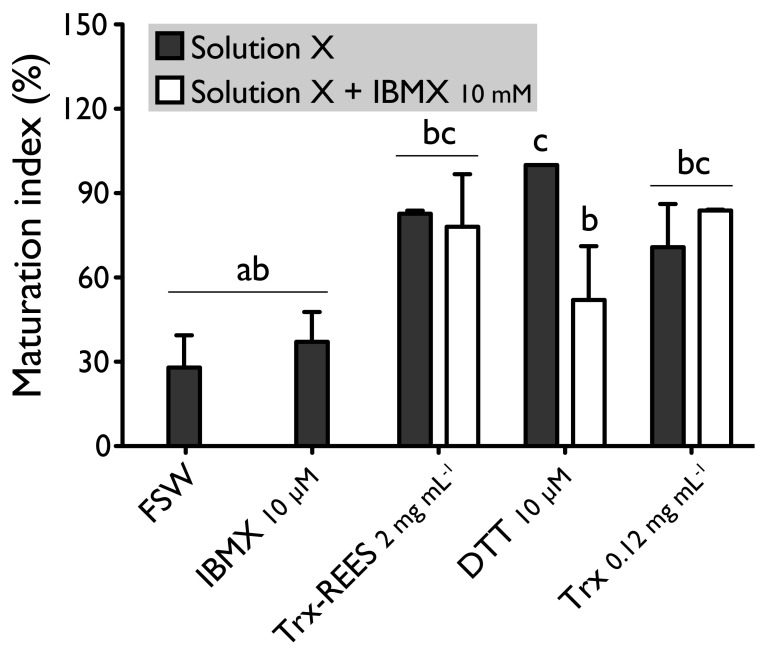
Effect of IBMX (10 μM), on *H. tubulosa* oocyte maturation, induced by Trx-REES (2 mg mL^−1^), DTT (10 mM) or thioredoxin from *E. coli* (Trx, 0.12 mg mL^−1^). Values are means of maturation index ± SD (*n* = 3 individuals). Means sharing at least one letter are not significantly different (T_Tukey_ ≥ 0.05).

We also tested the thioredoxin from *E. coli* with hypoxanthine (1 mM) (Figure 4) and IBMX (10μM) (Figure 5). Thioredoxin from *E. coli* effectively induces oocyte maturation despite the presence of hypoxanthine (1 mM) and IBMX (10 μM) as results with REES and contrarily to the results obtained with DTT. MI in hypoxanthine-DTT (42.2% ± 7.0%) and in IBMX-DTT (52.0% ± 19.1%) was significantly similar to MI in sea water (Hypoxanthine: 32.2% ± 8.6; IBMX: 27.9% ± 11.5%), to MI in hypoxanthine (36.4% ± 9.7%) (Figure 4) or MI in IBMX (37.1% ± 10.6%) (Figure 5). However, Trx from *E. coli* effectively induces oocyte maturation despite the presence of hypoxanthine (1mM) (Figure 4) and IBMX (10μM) (Figure 5). In this case, the MI in the hypoxanthine-Trx from *E. coli* (68.5% ± 5.3%) and IBMX-Trx from *E. coli* (83.8% ± 0.3%) solutions were the similar to MI in positives controls [Hypoxanthine: DTT: 100% ± 0%; Trx-REES: 86.5% ± 1.4%; Hypoxanthine-Trx-REES: 71.1% ± 17.9%; Thioredoxin: 75.8% ± 1.2% (Figure 4)–IBMX: DTT: 100% ± 0%; Trx-REES: 82.7% ± 1.1%; IBMX-Trx-REES: 78.0% ± 18.6%; Thioredoxin: 70.8% ± 15.4% (Figure 5)].

**Figure 6:**
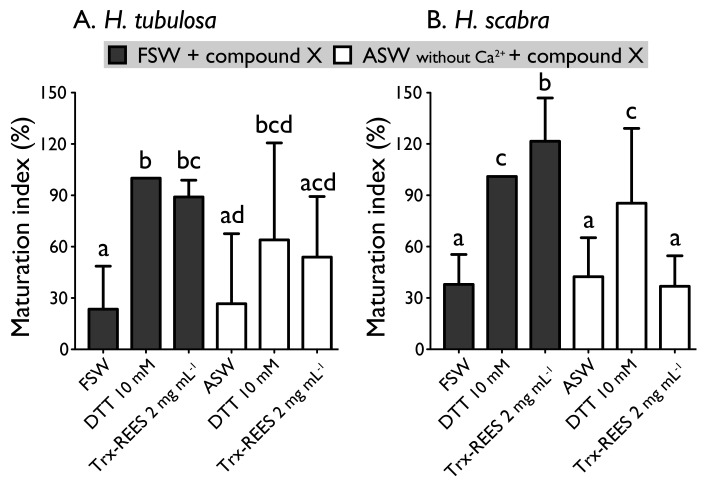
Effect of the Trx-REES and DTT to induce *H. tubulosa* (**A**) and *H. scabra* (**B**) oocyte maturation in filtered sea water (FSW) or in artificial sea water (ASW) without Ca^2+^. Values are means of maturation index ± SD (*n* = 3 individuals). Means sharing at least one letter are not significantly different (T_Tukey_ ≥ 0.05).

Figure 6 shows the MI obtained with oocytes of *H. scabra* and *H. tubulosa* incubated in ASW without Ca^2+^ with or without the inducers (DTT or REES). Exposure of oocytes in ASW without Ca^2+^ completely inhibited REES effect on *H. scabra* oocytes and considerably decrease REES effect on *H. tubulosa* oocytes. The corresponding MI for *H. scabra* (ASW+REES, 36.5% ± 17.7%) and for *H. tubulosa* (ASW+REES, 53.9% ± 35.4%) were significantly the same than MI in FSW (for *H. scabra*: 37.6% ± 17.3%; for *H. tubulosa*: 23.5% ± 25.1%) or in ASW (for *H. scabra*: 42.0% ± 22.6%; for *H. tubulosa*: 26.6% ± 40.9%) (Figure 6). DTT effect in ASW decreased but was not inhibited: the MI obtained with *H. scabra* oocytes (MI = 84.6% ± 43.3%) and *H. tubulosa* oocytes (64.0% ± 56.6%) were not significantly different than the MI obtained when DTT was used in FSW (100% ± 0%) (Figure 6).

## 4. Discussion

**Figure 7:**
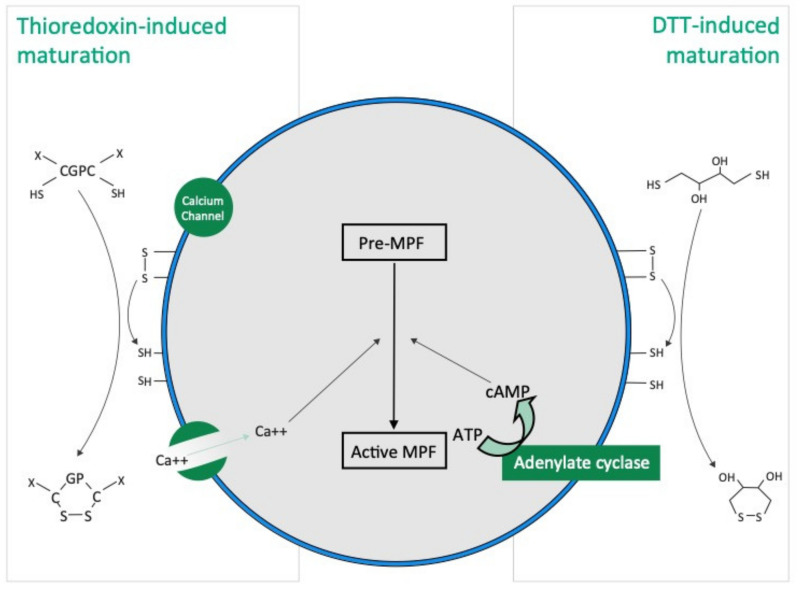
Hypothetical TRX and DTT induced oocyte maturation pathways based on the present study and the literature. DTT acts as a reducing agent by catalysing the reduction of disulphide bonds (R-SS-R), increasing the presence of sulfhydryl groups (i.e., R-SH), in hypothetical membrane proteins present at the surface of oocyte. Thioredoxins have a “CGPC” catalytic site and the presence of the two cysteines allows the reduction of disulphide bonds in hypothetical membrane proteins present at the surface of oocyte.

Our results show that the mode of action of thioredoxin in holothuroid oocyte maturation is different from that of DTT. DTT, also called Cleland’s reagent [33], is a small synthetic molecule which acts as a reducing agent by catalysing the reduction of disulphide bonds (i.e., R-SS-R) from substrates with which they interact (Figure 7). DDT is cyclic in the oxidised state [33]. In asteroids, DTT has been suggested to mimic 1 MeAd (i.e., the natural asteroid MIS) whose action increases the sulfhydryl concentration (i.e., R-SH) at the surface of oocyte before maturation [34]. The thioredoxin that we identified from rough extracts of echinoid spawns and that induce holothuroid oocyte maturation, the Trx-REES, is a 18 kDa natural protein [28]. Thioredoxins are folded proteins where the fold consists of five β-strands surrounded by α-helices [35]. All thioredoxins have a CGPC catalytic site where the presence of the two cysteines allows the reduction of disulphide bonds of various substrates. The catalytic CGPC motif is located on the surface of the protein in a short segment at the amino-end of the α2-helix [35]. Unlike DTT, Thioredoxins are ubiquitous molecules found in bacteria, plants and animals, both intracellularly (in their cytosol, nucleus, mitochondria) and extracellularly [29]. In holothuroids, a thioredoxin called Aj-Trx has been highlighted in the longitudinal muscles, the body wall, the coelomocytes, the digestive tract, the respiratory trees and the podia of the species *A. japonicus* (the gonads were not investigated) [36]. Aj-Trx is 38 kDa, is thought to be intracellular and has the typical catalytic site of thioredoxins [36]. With regard to *H. tubulosa* and *H. scabra* used in our experiments, and in view of the structure of Trx-REES, it is reasonable to think that it acts by reducing the disulphide bonds present in proteins located on the surface of oocytes (Figure 7). The structure of Trx-REES being very different from DTT we suggest that the interactions at the surface of oocytes are also different leading to the two non-identical activation pathways of oocyte maturation that we observed (Figure 7). In parallel, it is of interest to pinpoint that we have no experimental data to make assumptions about potential targets (extra- or intracellular) of the thioredoxins in the context of the sea cucumber oocytes. In the literature, mammalian thioredoxins have been shown to interact with a large number of target proteins to maintain a reducing environment (apoptosis signal-regulating kinase 1, Trx interacting protein, and phosphatase and tensin homolog…) [37].

In holothuroids, a maturation-inducing substance (MIS) has not been identified so far. Smiley isolated 2-methyl 8-amino-adenine from ovaries of *Apostichopus californicus*, and he assumed that this compound is the holothuroid MIS, but there was no direct evidence to support this assumption [38]. To find a substance able to induce in vitro holothuroid oocyte maturation, numerous molecules inducing asteroid oocyte maturation were tested on holothuroid oocytes: 1-Methyladenine (1-MeA) [24,27]; Dithiothreitol (DTT) [27,34]; L-cystein [24,27]; dimercapto-propanol (BAL) [27,39]; (8R)-hydroxyeicosatetraenoic acid (8-HEPE) [26] and Trx-REES [27]. Some of these products are ineffectual: 1-MeA, BAL and 8-HEPE cause no maturation [26,27]. DTT was with Trx-REES, the most effective compound [25,27,39]; however, it induces poor fertility and major larval abnormalities [27,39,40]. None of these deleterious effects were recorded when using Trx-REES [28]. Trx-REES is certainly not a GSS because our results show that it can induce oocyte maturation in follicle-enclosed oocytes as well as in follicle-free oocytes (by definition, a GSS is a substance that induces GVBD by acting on follicle cells [39]).

MIS are produced by follicle cells and act on receptors localised on the oocyte plasma membrane to transfer the signal into the cytoplasm of oocyte [41]. Trx-REES is also certainly not the MIS of holothuroids as it comes from an extract of echinoid samples, but it is possible that another thioredoxin would be the MIS in holothuroids as thioredoxins (i) are found in echinoid ovaries and in holothuroid tissues (though not yet searched and thus not found in holothuroid follicle cells), (ii) act at the surface of oocytes, and (iii) induce oocyte maturation.

In the present experiments, forskolin, hypoxanthine and IBMX, compounds that elevate the intracellular level of cAMP, inhibit oocyte maturation induced by DTT. This confirms the results described by Karaseva and Khotimchenko on holothuroids [26]. Despite the presence of forskolin, hypoxanthine and IBMX, REES induces oocyte maturation, contrary to DTT. Yet, thioredoxin from *E. coli* was also able to induce oocyte maturation in the presence of hypoxanthine and IBMX. Our results demonstrate thus that thioredoxin action is cAMP independent. cAMP is involved in a variety of cellular activation processes through cAMP-dependent protein kinases and, at least in mammals, it is well established that cAMP maintains the oocyte in meiotic arrest and that reinitiation of meiosis is subsequent to a drop in intraoocyte concentrations of cAMP (see [42] for review). More specifically in asteroids, a decrease of cAMP is also triggered during oocyte maturation by 1MeAd [43], which binds to an unidentified receptor to separate subunit of protein G and initiate a cascade that activates the MPF (i.e., CDK1 and Cyclin) and induce GVBD (see [44] for review).

It is well established that calcium is involved in the physiology of the oocyte from oogenesis to maturation and fertilisation. The calcium rise in the cell occurs by means of two principal mechanisms: the efflux from the stores via ligand-gated channels on organelle membranes, and the entry through ion channels in the plasma membrane. During oocyte maturation, these two mechanisms can occur together in some animals while in some others, only the second mechanism involving only channels on intracellular organelles are involved. This seems to be the case for asteroids where, when enclosed in their follicles, asteroid oocytes undergo maturation only in the presence of Ca^2+^, whereas no maturation occurs when the follicles are isolated in calcium free seawater [45]. This phenomenon, called “spontaneous maturation,” was later found to be due to an action of Ca^2+^ on the follicle cells rather than on the oocyte itself with calcium miming GSS in stimulating the release of 1-MeAd by the follicle [45]. In the present experiments, DTT-induced oocyte maturation seems to be independent from the extracellular calcium. On the other hand, the role of ion channels and extracellular calcium has been demonstrated in the meiotic resumption of oocytes in molluscs, ascidians, amphibians and mammals [46]. This is also the case in our experiments, where thioredoxin-induced oocyte maturation appears to be dependent of extracellular calcium. We did not investigate if calcium acts on follicle cells or oocytes, but the fact that thioredoxin acts at the surface of oocytes may suggest that this action could activate voltage-dependent calcium channels.

Roscovitine is potent at inhibiting p34^cdc2^ the cyclin-dependent kinases component of MPF. Roscovitine reversibly arrests asteroid and echinoid oocytes in late prophase [47]. It also blocks progesterone-induced oocytes maturation of *Xenopus* oocytes by inhibition of MPF activity [48]. Results presented in this paper show that holothuroid oocyte maturation started by REES or DTT is inhibited when oocytes are in contact with roscovitine, and thus suggests that maturation induced by REES and DTT requires the activation of p34^cdc2^ a principal compound of MPF.

MPF activity always appears to be associated with a high level of phosphorylation, as observed during meiosis reinitiation in amphibians [49], mammals [50] and asteroids [51,52]. 6-dimethyl-aminopurine (6-DMAP) was frequently used as a non-specific kinase inhibitor without inhibiting protein synthesis. We found that proteins phosphorylation seems to be involved in the resumption of holothuroid meiosis, which was activated by REES or DTT as our results show that use of 6-DMAP blocks the meiotic cell cycle. The results obtained using the genistein and the lavendustin A, two inhibitors of tyrosine kinase, showed that these kinases are not involved in holothuroid oocyte maturation as none of these molecules inhibited oocyte maturation in presence of REES or DTT. Results obtained by 6-DMAP, genistein and lavendustin lead to the hypothesis that only serine/threonine kinases would be involved in the MPF activation by Trx-REES or DTT.

According to the species, oocyte maturation requires or not a protein synthesis. The time of this synthesis makes control for the entrance into maturation via the synthesis of elements necessary to obtain active MPF. This was the case for the oocytes of cows [53], sows [54] and primates [55], asteroids [56], mice [57] and *Xenopus* [58], but under an inactive form. MPF become functional only after phosphorylation or/and dephosphorylation [56,59]. A synthesis of cyclin B was detected in early metaphase I in oocytes of asteroids and *Xenopus* [60,61]. For holothuroids, the use of cycloheximide, an inhibitor of protein synthesis, blocks the resumption of meiosis in both DTT- and REES-induced maturations suggesting that some protein syntheses are necessary for the resumption of their meiosis.

## 5. Conclusions

Our experiments show that holothuroid oocyte maturation can be activated by two reducing agents, DTT and thioredoxins, which act by reducing the disulphide bonds to sulfhydryl groups at the surface of oocytes. The maturation induced by DTT is dependent on cAMP and does not require extracellular calcium while the maturation induced by thioredoxin is on the contrary independent of cAMP and requires the presence of calcium in seawater. Thioredoxins being a natural product found in echinoderms, future experiments will have to show whether one of them is the natural inducer (MIS) of holothuroid oocyte maturation.

These results are of interest for the holothuriculture field. Indeed, a crucial step of this activity is to obtain a maximum number of fertilised eggs. Generally, gamete laying is induced by thermal shocks applied on genitors; however, this method lacks efficiency and only a small fraction of genitors effectively laid their gametes. The use of REES/thioredoxin to induce maturation of oocytes taken by dissections of ovaries opened a new avenue to obtain embryos (fertilised in vitro) in sea cucumber hatchery [27,28].

## Figures and Tables

**Table 1:** Pharmacological agents with their specificity and their effect on oocytes maturation. All pharmacological agents were obtained at Sigma-Aldrich. Legend. DMSO: Dimethylsulfoxid. At higher concentrations the products became toxic for oocytes, or they were not soluble. The results presented in the present paper only show those obtained for the smallest active and non-toxic concentration.

Pharmacological Agent	Action	Solubilisation	Tested Concentration	Active and Non-Toxic Concentration
Forskolin	Activator of adenylate cyclase	DMSO	1; 5; 10 µM	10 µM
3-isobutyl-1-methylxanthine (IBMX)	Inhibitor of phosphodiesterases	DMSO	0.1; 1; 10; 100 µM	10 µM
Hypoxanthin	Inhibitor of phosphodiesterases	tris 25 mM pH 7.2 buffer	0.1; 1; 10; 100; 1000 µM	1 mM
6-dimethyl-aminopurine (6-DMAP)	Inhibitor of serine threonine kinase protein	tris 25 mM pH 7.2 buffer	50; 100; 150; 200; 250; 300; 350; 400 mM	300 mM
Lavendustin	Inhibitor of tyrosine kinase protein	DMSO	1; 10; 100 µM	10 µM
Genistein	Inhibitor of tyrosine kinase protein	DMSO	1; 10; 100 µM	10 µM
Roscovitine	Inhibitor of cdk1	DMSO	0.1; 1; 10 µM	10 µM
Cycloheximide	Inhibitor of protein synthesis	tris 25 mM pH 7.2 buffer	5; 10; 50 mM	50 mM

## Data Availability

Data is contained within the article.
